# Case report: fainting during acupuncture stimulation at acupuncture point LI4

**DOI:** 10.1186/s12906-017-1656-9

**Published:** 2017-03-09

**Authors:** O sang Kwon, Kwang-Ho Choi, Junbeom Kim, Seong Jin Cho, Suk-Yun Kang, Ji-Young Moon, Yeon Hee Ryu

**Affiliations:** KM Fundamental Research Division, Korea Institute of Oriental Medicine, Daejeon, Korea

**Keywords:** Fainting during acupuncture, Acupuncture, Fainting, EEG, sLoreta

## Abstract

**Background:**

Fainting is one of the major adverse events that can occur as a result of acupuncture treatment. However, the observation of changes in biological parameters is rarely available when fainting occurs. In this case report, we could observe changes in the electroencephalogram (EEG) in a participant who fainted while participating in a clinical trial aiming to observe a relationship between acupuncture stimulation at LI4 acupuncture point and EEG in healthy adults.

**Case presentation:**

The EEG pattern of participant changed twice. The first change was in response to the acupuncture needle insertion, and the second change occurred during fainting. Both changes consisted of a burst in EEG amplitude, but the pattern of details was different. Multiple areas of the cortex were activated, and the increased ratio of the γ wave was not observed during fainting. While acupuncture needle insertion, only the sensory cortex were activated and increased the ratio of the γ wave.

**Conclusions:**

This single case is presented to improve the understanding of fainting during acupuncture as an adverse event and to explore the mechanism of acupuncture treatment, despite the absence of statistics and repeatability. This information can provide a new viewpoint about the mechanism of acupuncture treatment and the possibility of new techniques based on acupuncture.

## Background

The efficacy, economical feasibility and safety of acupuncture treatment have been demonstrated in thousands of clinical studies. Acupuncture treatment can replace or reduce the dosage of the drugs such as amitriptyline [1], taxane [2] or morphine [3] to prevent adverse event that are prescribed to patients to treat various disease including chronic pain. This replacement reduces the adverse effects of drugs, however, acupuncture treatment may also be associated with minor and less critical adverse effects.

Pain and bleeding are reviewed as the most common adverse events (AEs) of acupuncture treatment from 73 case reports and 14 case series [4]. In addition to these mild AEs, fainting, stroke, haemorrhage or traumatic injuries were reported as serious AEs and led to death in some cases [4]. However, a more recent survey indicated that acupuncture clinics and the potential of severe AEs are rare [5].

Fainting is a common AE which contains dizziness, perspiration and syncope as a symptom [6], during acupuncture treatment and has not been classified as a critical AE because fainting does not kill the patient or have long-term effects, although it does cause some patients to avoid future acupuncture treatments because of the traumatic experience. However, some researchers have proposed that fainting can be used to access the mechanism of acupuncture treatment in the brain. The mechanism and cause of fainting during acupuncture treatment have not been closely examined because researchers cannot induce participants to faint.

This case occurred as an adverse event during a clinical trial designed to observe changes in EEG patterns affected by acupuncture treatment. EEG signals were recorded in the pre-stage before acupuncture needle insertion, and recording was discontinued when the participant fell down. The EEG pattern during fainting was collected until the participant fall down, and we present the data of this single case herein.

## Case presentation

### Participant information

Age: 20

Gender: female

Height: 160.0 cm

Weight: 41.0 kg

Body temperature: 37.4 °C

Average blood pressure: 106/61 mmHg

Marriage: none

Job: student (college)

Diet: regular

Exercise: none

Smoking: none

Drinking: 0.5 bottle (beer)/week

Specific disease: none

Medical history: no specific medical history

### Adverse event

Participant were participated in the clinical trial to observe the effect of acupuncture stimulation at the electroencephalogram (EEG) and peripheral nerve system in male and female adults at the Daejeon Korean medicine hospital of Daejeon university (KCT0001871). The adverse event occurred during visit 2 (2015.05.20). The participant arrived at 10 am, and her behaviour and language were normal. The participant was prepared for measurements with a multichannel EEG (ActiveTwo, BIOSEMI, Netherland) and was placed in a seated position on a chair. Electrodes were attached, and the strength of the electrical stimulation was calibrated. A sterilised single-use acupuncture needle (0.3 × 30 mm, Dongbang medical, Korea) was inserted into the acupuncture point large intestine 4 (LI4, Hapgok) of right hand of participant approximately in depth of 1.8 cm by licensed KMD who has a 10 year’s career and 4 year of the career was as a assistant teacher of acupuncture practice at the university, after 30 min of rest. The EEG data were recorded for approximately 5 min before the acupuncture needle was inserted, and researchers noticed the fainting approximately 4 min after needle insertion. The participant did not report any symptoms or uncomfortable feelings. The researchers heard the sound of some objects falling down and saw participant falling at that time. The EEG electrodes were separated from the participant’s body due to the impact of falling down.

### Subsequent observations

We placed the participant on the bed in a supine position. The participant recovered before 1 min had passed. The participant drank a cup of warm water and rested for 30 min while lying on the bed.

The participant’s condition was observed at both 7 and 30 days after fainting. The participant did not report any uncomfortable feeling or poor condition. She also did not show any traumatic reaction or negative opinion about acupuncture treatment and was willing to receive acupuncture treatment again.

During the first interview, the participant admitted that she had been on a diet while participating in the test.

### Analysis

Frequency analysis using Fourier transformation was performed to quantitatively analyse the changes in EEG activity. A window of 2 s (75% sliding window) was applied to the full-time data. The ratio was normalized as the ratio of the total power at each window after adding the absolute power of each frequency band (ε: 5–8 Hz, α: 9–12 Hz, β: 13–30 Hz, γ: over 30 Hz).

Standard low-resonance electromagnetic tomography analysis (sLoreta) [7, 8] was performed using a LORETA-KEY program available at: http://www.uzh.ch/keyinst/loreta.htm.

### EEG wavelength amplitude

**Fig. 1:**
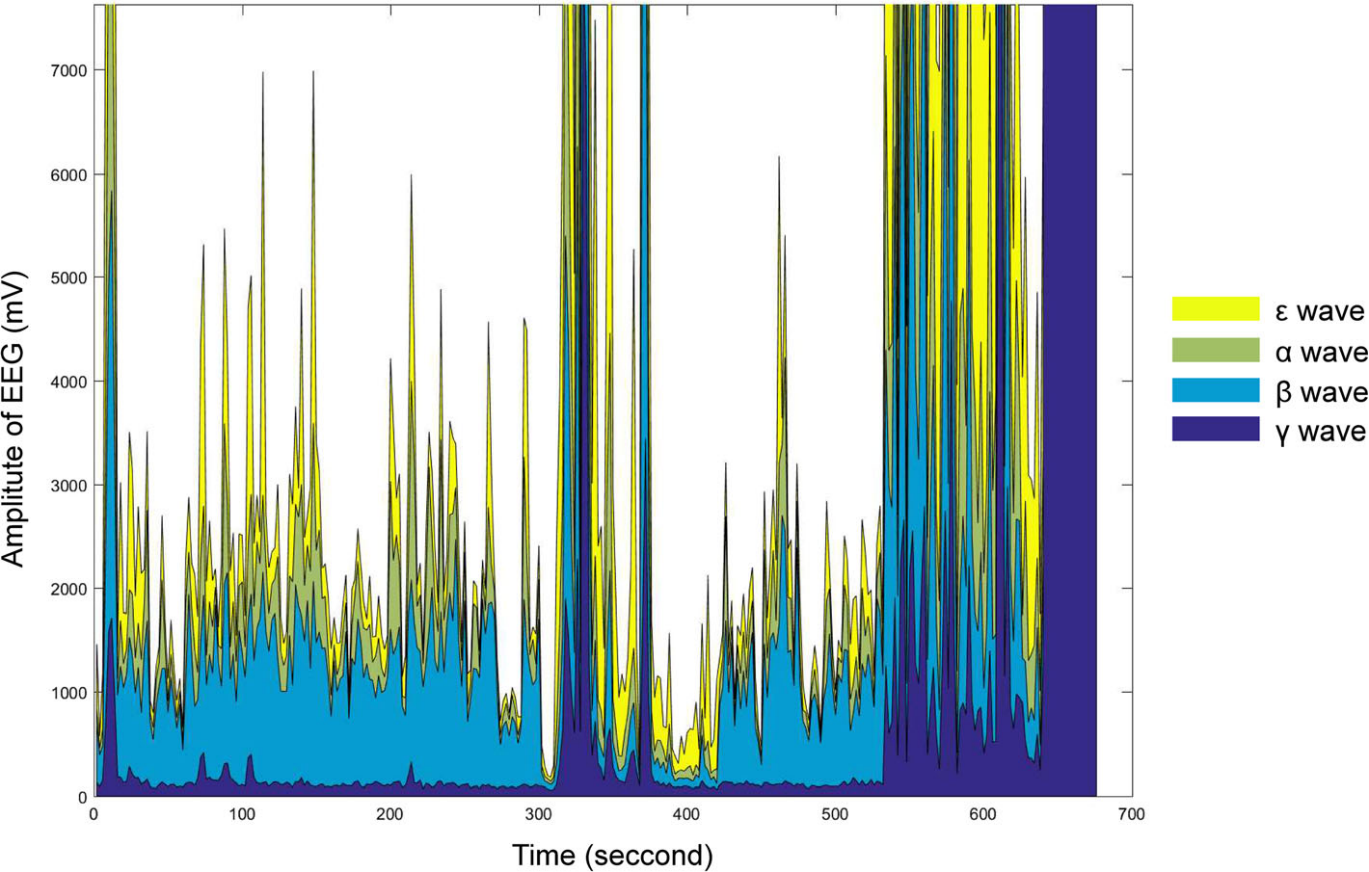
Changes in the absolute power of EEG bands. The acupuncture needle was inserted at approximately 300 s (*red vertical line*), and fainting was observed at approximately 540 s. The EEG amplitude rapidly increased twice: once at the time of needle insertion and once at the time of fainting

**Fig. 2:**
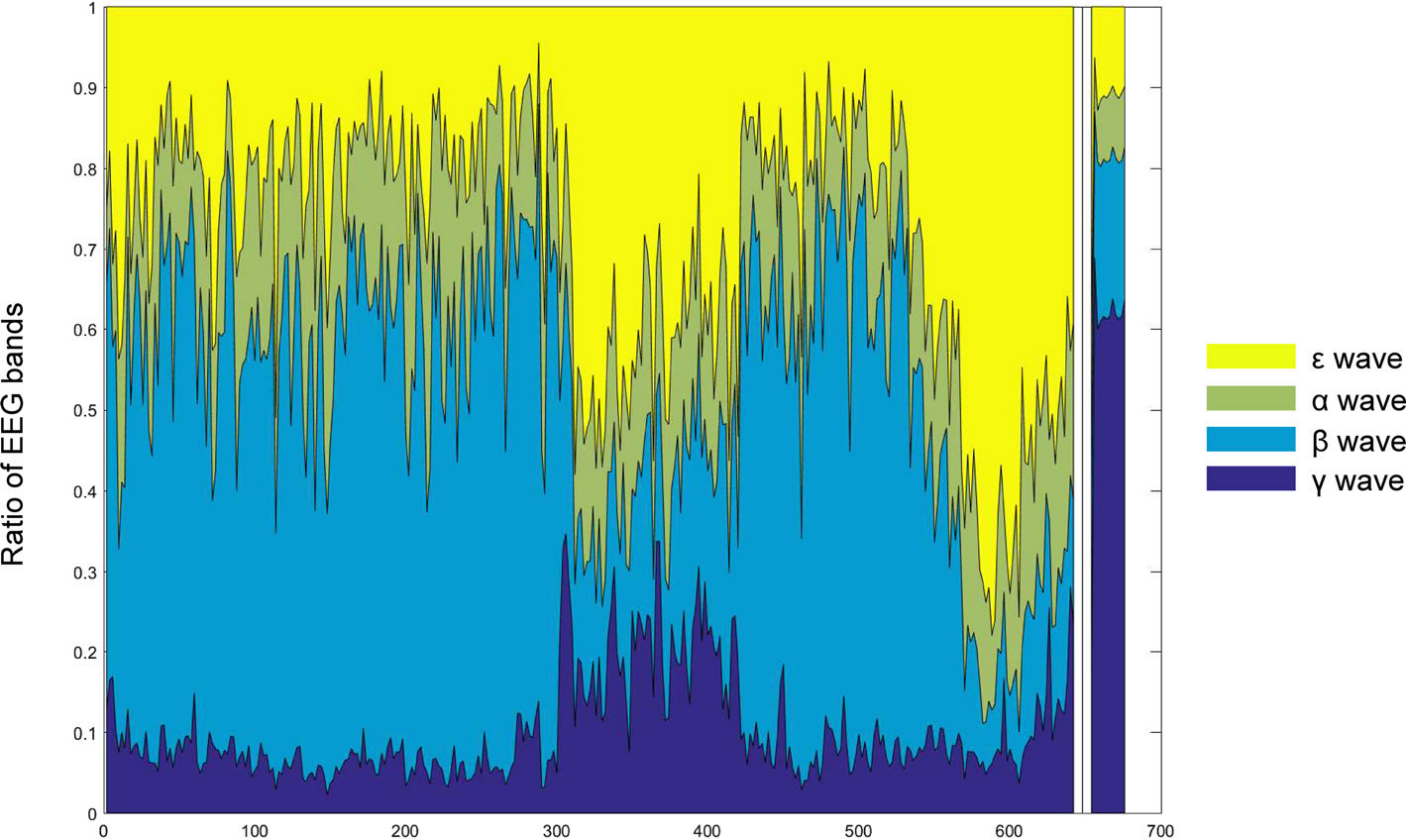
Changes in ratio between each EEG band. Both activity bursts showed an increase in the ε wave ratio. The ratio of the γ wave did not significantly increase when the patient fainted, but it did increase significantly when the acupuncture needle was inserted

**Fig. 3:**
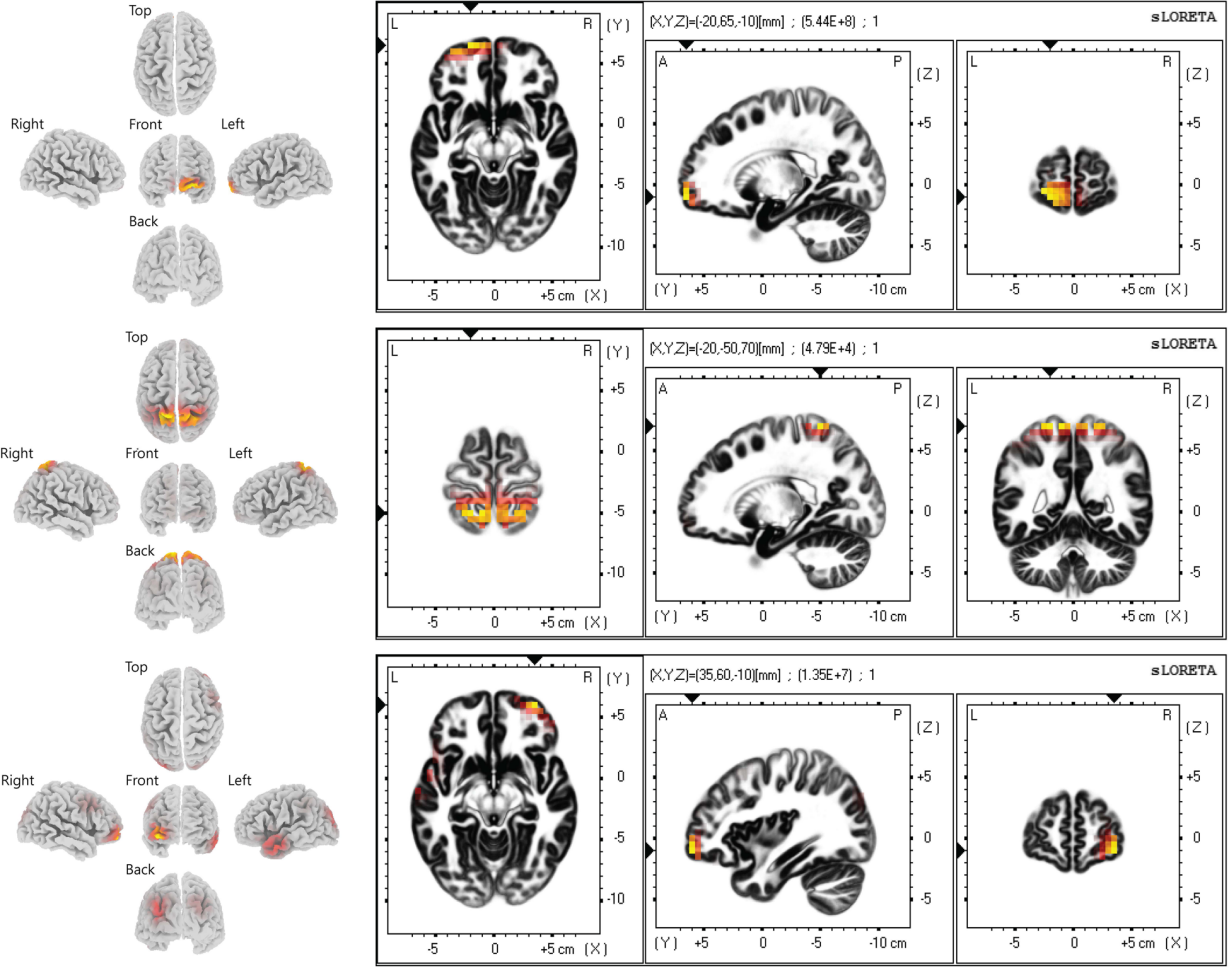
Cortical areas activated at each stage. *Coloured spaces* indicate an increase in neuronal activity. The *yellow* colour indicates a more active area than a red-coloured area. *Upper*: baseline activity before acupuncture needle insertion. *Middle*: the first burst when the acupuncture needle inserted; the sensory cortex was activated. *Lower*: the second burst when the participant fainted; activation occurred in various areas

The absolute power of the EEG signal increased twice during the procedure. The first burst was observed after the acupuncture needle was inserted (300 s). The next burst was observed after the participant fainted (540 s). An increase in the γ wave was observed (Fig. 1). The ratios of the ε, α, β and γ wave bands showed a specific pattern at the same time of the bursts. The ratios of the ε and γ waves increased significantly, and the ratio of the β wave decreased significantly (Figs. 2 and 3).

### EEG activity in the brain

EEG activity in the brain was analysed using the Brodmann area (BA) [9]. The orbitofrontal cortex (BA11) was activated before acupuncture treatment. The medial part of the primary sensory cortex (BA3, 1 and 2) and the somatosensory association cortex (BA5) were activated after the acupuncture needle was inserted. While the patient was fainting, the EEG signal was activated at various areas: the dorsolateral prefrontal cortex (DLPFC, BA46,9) or insula cortex (BA16); pars orbitalis (BA 47); part of the secondary visual cortex (V2, BA18) and the associative visual cortex (V3,V4,V5, BA19); and the middle temporal gyrus (BA21).

## Discussion and Conclusion

Fainting during acupuncture treatment is one of the most common adverse effects that occurs at the acupuncture clinic [4] and is believed by some researchers to be related to the mechanisms of acupuncture treatment. However, studies on fainting during acupuncture have not been performed because of ethical and technical issues. Thus, fainting during acupuncture in a clinical trial, particularly a brain imaging study, can be a great opportunity to observe what happens as fainting occurs. In this study, we had the opportunity to observe EEG patterns during fainting and present the data herein to enlarge our experience and understanding the relationship between the brain and acupuncture.

Before acupuncture needle injection, the amplitude of the EEG did not show a specific pattern. EEG activity was detected in the orbitofrontal cortex (BA11), which is known to be related to the reward system [10]. We hypothesized that the patient was anxious and afraid of acupuncture following her interview; this was her first experience with acupuncture.

The absolute power of the EEG signal significantly increased twice, and the patterns of the two bursts were different. The ratio and absolute power of the γ wave, which is related to the somatosensory [11] area and perception [12], increased significantly and appeared to modulate the stimulation from the acupuncture needle insertion. This analysis was supported by sLoreta location analysis, which showed EEG activity in the medial part of the primary sensory cortex (BA3, 1 and 2) and the somatosensory association cortex (BA5), thus which indicating stimulation of the forearm or hand. The increase of EEG amplitude was observed at prior studies [13, 14] and even very significantly increasing of the amplitude also observed [15], however, in some study, we could find that most of channels are not changed [1].

The second burst showed a different pattern. The ratio and absolute power of the γ wave did not increase when the burst started, whereas the first burst showed an increase in the γ wave at the start of the burst. The sLoreta analysis also indicated different locations of activation. The amplitude of the brain waves increased at various locations of cortex, such as the DLPFC, the insula cortex, the pars orbitalis, part of the secondary/associative visual cortex and the middle temporal gyrus. Activation of the DLPFC and the insula cortex during acupuncture treatment has been reported by various researchers [16, 17]. However, the other areas were not reported to have a major role and did not have any relationship with the efficacy area of LI4. Thus, we considered that additional excitation at these areas may provoke fainting, although the mechanism and connection between these area is not clear.

In the later interview, participant reported that she had never experienced fainting or syncope. Participant’s average systolic/diastolic blood pressure before the test was 106/61 and we concluded it as a normal range and not enough for fainting regarding her body mass index. She reported she was not fasted although she was trying to lose her weight. There was no patients of epilepsy, amyotrophic lateral sclerosis or other genetic neural disease in her family according herself and she had never experienced fainting before the test in accordance with her statement. We also asked the participant to contact us when she experience fainting or similar situation again and we could get any message from her for more than one and half years. We concluded that her physical statement such as blood pressure, body weight or etc. stayed at the low but normal range and may work without any problem before, however, those statement may affected her to faint when we induced acupuncture stimulation.

This case is the first reported case of EEG changes observed while fainting caused by acupuncture stimulation and comparison or statistical analysis could not performed. Thus, this one case cannot reveal the parameters or mechanisms of fainting during acupuncture treatment. However, fainting cannot be intentionally induced by acupuncture, and this accidental case is considered to be the first report about what occurs in the brain when people faint during acupuncture treatment.

## Abbreviations

AEs: Adverse events
BA: Brodmann area
EEG: Electroencephalogram
sLoreta: Standard low-resonance electromagnetic tomography analysis
